# Real-world evidence from the first online healthcare analytics platform—Livingstone. Validation of its descriptive epidemiology module

**DOI:** 10.1371/journal.pdig.0000310

**Published:** 2023-07-25

**Authors:** Benjamin R. Heywood, Christopher Ll. Morgan, Thomas R. Berni, Darren R. Summers, Bethan I. Jones, Sara Jenkins-Jones, Sarah E. Holden, Lauren D. Riddick, Harry Fisher, James D. Bateman, Christian A. Bannister, John Threlfall, Aron Buxton, Christopher P. Shepherd, Elgan R. Mathias, Rhiannon K. Thomason, Ellen Hubbuck, Craig J. Currie

**Affiliations:** 1 Human Data Sciences, Cardiff, United Kingdom; 2 School of Mathematics, Cardiff University, Cardiff, United Kingdom; 3 Human Data Sciences, North Carolina, United States of America; 4 School of Computing, Cardiff University, Cardiff, United Kingdom; 5 Division of Population Medicine, School of Medicine, Cardiff University, Cardiff, United Kingdom; Duke-NUS Medical School, SINGAPORE

## Abstract

Incidence and prevalence are key epidemiological determinants characterizing the quantum of a disease. We compared incidence and prevalence estimates derived automatically from the first ever online, essentially real-time, healthcare analytics platform—Livingstone—against findings from comparable peer-reviewed studies in order to validate the descriptive epidemiology module. The source of routine NHS data for Livingstone was the Clinical Practice Research Datalink (CPRD). After applying a general search strategy looking for any disease or condition, 76 relevant studies were first retrieved, of which 10 met pre-specified inclusion and exclusion criteria. Findings reported in these studies were compared with estimates produced automatically by Livingstone. The published reports described elements of the epidemiology of 14 diseases or conditions. Lin’s concordance correlation coefficient (CCC) was used to evaluate the concordance between findings from Livingstone and those detailed in the published studies. The concordance of incidence values in the final year reported by each study versus Livingstone was 0.96 (95% CI: 0.89–0.98), whilst for all annual incidence values the concordance was 0.93 (0.91–0.94). For prevalence, concordance for the final annual prevalence reported in each study versus Livingstone was 1.00 (0.99–1.00) and for all reported annual prevalence values, the concordance was 0.93 (0.90–0.95). The concordance between Livingstone and the latest published findings was near perfect for prevalence and substantial for incidence. For the first time, it is now possible to automatically generate reliable descriptive epidemiology from routine health records, and in near-real time. Livingstone provides the first mechanism to rapidly generate standardised, descriptive epidemiology for all clinical events from real world data.

## Author summary

Analyses of large datasets from patients’ care are now common. These studies are complex though. They normally require a team of people with a range of advanced skills. This is particularly true of UK NHS data. Because of these various hurdles, until now, automation of these types of healthcare study has never happened. Here we report the first automated analysis platform that does this, called Livingstone. Livingstone very quickly can carry out some types of health-related studies that has typically taken 12 to 24 months to complete. Here we report how we checked the output numbers generated by Livingstone versus corresponding numbers published in peer reviewed scientific studies. Livingstone performed extremely well.

## Introduction

Two of the most commonly used metrics characterising the descriptive epidemiology of any disease, condition or clinical intervention are their incidence and prevalence. Rassen and colleagues explained some of the many technical challenges involved in deriving these parameters for chronic diseases [1]. Lifelong conditions are technically the easiest to characterise because once an individual is diagnosed with a disease they remain in the pool of prevalent cases, and only their first recorded event is incident. More complicated to characterise are the incidence and prevalence of acute or chronic conditions that do not have a lifelong duration. For instance, in determining the epidemiology of acute cough, it is not obvious whether two cough diagnoses recorded 12 weeks apart are two distinct, incident events or represent a chronic cough [2]. This can lead to differing estimates where researchers have used different case definitions. Accounting for these considerations in an automated analytical system to produce reliable, replicable descriptive epidemiology requires standardised methods for eliciting and capturing user requirements, plus algorithmic decision rules.

The most common method of determining disease epidemiology is by analysis of routine health records, now commonly knows as real-world data (RWD). These records can come from various healthcare provider or user sources but the most widely used are from general practice or from hospital admissions and outpatient attendances. Ideally these should be record-linked.

Currently, three main clinical computer systems are used in UK primary care to manage patient records: Vision, Egton Medical Information Systems (EMIS) and SystmOne. These systems record clinical activity in different ways and use differing data models. The collective analysis of data from more than one of these systems demands a lot of care. A further constraint to delivering reliable and useful epidemiological outputs are the skills, experience, time and financial costs of research. Epidemiologists from Cambridge University stated recently that it can cost almost £200,000 and take up to two years to carry out this type of study using routine NHS data from sources such as the Clinical Practice Research Datalink (CPRD) [3].

Adding to the analytical complexities and data quality issues, the computational challenges of developing an automated, analytical platform for this purpose are manyfold. For instance, incidence can be determined at any time from the inception of the data source to the last data-collection point. Patients’ case histories often change rapidly, but automation requires that these complex calculations have to be computed contemporaneously with the selected time-points for the duration of the data source, once factors such as the target study group, time point(s) of interest, and other study characteristics have been user-defined.

To our knowledge, no automated system has yet been devised that can determine these descriptive epidemiological metrics from routine healthcare data; for all diseases or other clinical events, for phenotypic sub-groups, over an extended observation period, and in near-real time; meaning that it can take a few minutes to process. Due largely to the evolution of cloud computing, it has only now become possible to carry out these complicated calculations on these large and complex datasets. The purpose of this study was to validate the epidemiological outputs from Livingstone—the first such analytical platform—by comparing its automated incidence and prevalence values with analogous peer-reviewed findings.

## Methods

### Analytical platform

Livingstone is a cloud-based analytics platform that analyses complex healthcare data in near-real time [4]. Livingstone presents technical and non-technical users with analytical tools enabling the rapid production of complex health intelligence. Livingstone allows the user to create code lists through browsable clinical dictionaries or to upload existing code lists. Such lists can then be used to define and select a study cohort, which may then be further refined, if necessary, based upon detailed real-time exploration of various patient characteristics. The final study cohort is then analysed by Livingstone to produce the epidemiological findings. A corresponding cost module is also available, calculating the resource use and financial costs of general practice contacts, prescribed drugs and devices, outpatient attendances and inpatient admissions. Other modules are either in development or planned.

The purpose of the epidemiological module integral to Livingstone is to compress a scientific study that would otherwise require a team of experienced investigators 12 to 18 months to complete, into only a few minutes. This is done by removing the need for essential researcher inputs such: data cleaning, data manipulation, code development, code checking and output validation.

### Data source

For these analyses, Livingstone used primary care data from CPRD. CPRD’s datasets, GOLD (Vision) and Aurum (EMIS), comprise longitudinal pseudonymised data from general practices in the UK [5,6]. This study used data to June 2021 from CPRD-Aurum, and data to July 2021 from CPRD-GOLD. Data were available for over 72 million people from approximately 2,500 general practices. Vision and EMIS are two of the most commonly used systems in the UK used to manage patients’ clinical records.

### Ethical approval

CPRD data are obtained under licence from the UK Medicines and Healthcare products Regulatory Agency. This study has received CPRD Research Data Governance approval (22_002001). RDG approval for this study was to validate a standardised algorithm to estimate the prevalence and incidence of selected conditions in the Clinical Practice Research Datalink. This algorithm underpins the Livingstone platform- the first such analytical platform that can generate automated incidence and prevalence values.

### Selection of published epidemiological data

A search of the US National Institutes of Health’s PubMed archive of biomedical literature was conducted to identify relevant studies containing incidence and prevalence statistics. The following search strategy was used, requiring all criteria to be satisfied:

1. The primary outcome was either prevalence or incidence based on title keywords
2. The data source was one or more of the four main UK primary care databases: The Health Improvement Network (THIN) [7], the General Practice Research Database (GPRD; CPRD’s precursor) [8], CPRD [8] or QResearch [9]
3. The date of publication was after the 1^st^ January 2016
4. Upon review, papers included relevant epidemiological statistics.
5. Findings were presented by individual calendar year(s) and not over a multi-year timeframe.

The diseases that were the subjects of the studies meeting our criteria were then analysed in Livingstone as individual disease cohorts.

### Case selection from Livingstone

The analyses of each disease cohort were conducted using combined GOLD and Aurum datasets. These data comprised male and female patients who were of acceptable research quality as defined by CPRD and had at least one day of registered follow-up. Cases from CPRD GOLD were required to have at least one day of up-to-standard (UTS) follow-up, a CPRD quality metric (not applied in CPRD Aurum) that takes into consideration practices’ death recording and continuity of data. This excluded 21% of cases. To avoid duplication, cases from CPRD GOLD whose GP practice subsequently migrated to CPRD Aurum were excluded from the combined data, as were cases registered in 29 practices in Aurum flagged as being duplicated.

The majority of the selected papers were accompanied by lists of clinical codes defining the disease(s) they reported. Where possible, patients were therefore selected from the combined CPRD data using codes that mapped directly to the published codes for the disease in question. Where the published codes were not exactly applicable to the CPRD data, these were mapped to the nearest applicable codes using our own algorithms, and where papers were not accompanied by code lists, we compiled our own.

The maximum observation period was 1^st^ January 2004 to 31^st^ December 2020. For each patient the start of CPRD follow-up was set as their practice registration date or, in CPRD GOLD, as the latter of their registration date or their practice’s UTS date. End of CPRD data follow-up was defined as the earliest of the patient’s transfer out from the practice (if applicable), their date of death (if applicable), or their practice’s final date of data-collection. The presentation date was defined as that of the patient’s first clinical record indicative of the relevant disease.

### Incident and prevalent populations

For chronic conditions, point prevalence was calculated. Patients in the disease cohort were eligible for inclusion in the point prevalence analysis if both their CPRD follow-up period and their exposure to the selected disease overlapped the midpoint (30^th^ June) of any calendar year. The denominator population for the prevalence analysis comprised all patients of acceptable research quality in the combined dataset having CPRD follow-up that overlapped the midpoint of a calendar year.

For acute conditions, period prevalence was calculated. Patients in the main cohort were eligible for inclusion in the period prevalence analysis if their CPRD follow-up period and their exposure to the condition overlapped any part of any calendar year in the observation period. The denominator population for the period prevalence analysis comprised all patients of acceptable research quality in the combined dataset having CPRD follow-up that overlapped the midpoint in the calendar year.

Patients in the disease cohort were eligible for inclusion in the incidence analysis if they had a presentation date within the CPRD follow-up period, with that incident record occurring 90 days or more after their registration date. For lifelong diseases, the patient was considered to be exposed to the condition until the end of CPRD follow-up. For all other diseases, a patient was considered to be exposed to the disease for the user-defined expected duration of that disease, and a record of the disease was considered to be incident if there was no other record of that disease in a preceding period commensurate with the user-defined disease duration. The denominator for the incidence analysis comprised the total registration period for all research-quality patients in the combined dataset having at least 90 days’ registration.

### Statistical methods

Incidence was calculated over the observation period using incident cases per calendar year as the numerator, and the aggregated, observed person-time per year in all registered, eligible patients as the denominator. For each year, person-time was calculated as the difference between the latest of 1^st^ January, the patient’s start of CPRD follow-up, and registration date plus 90 days, and the earliest of the onset of their specific disease event, 31^st^ December, and the patient’s end of CPRD follow-up. Incidence rates are presented for the UK overall.

Period and point prevalence values were calculated depending on whether the disease in question was acute, non-lifelong or chronic. Point prevalence was calculated at the midyear points (30^th^ June) over the observation period, as appropriate. For point prevalence, patients exposed to a disease at each midyear point formed the numerator. For period prevalence, patients exposed to an acute disease during the year comprised the numerator. The eligible CPRD population at each midyear formed the denominator. Prevalence was presented for the UK population overall.

Settings for disease chronicity and expected duration, and the clinical code lists used to select disease cohort members were defined before computing incidence and prevalence. Once the platform had produced incidence and prevalence findings based on each disease, we then compared these values with the published findings.

Lin’s concordance correlation coefficient (CCC) was calculated between values produced by Livingstone and the corresponding publications. This was conducted using the CCC command from the *DescTools* package of R statistical software. Lin’s CCC is robust when calculated on as few as 10 observations [10]. There are different interpretations of Lin’s CCC but the most robust recommendations are: <0.90, poor; 0.90 to 0.95, moderate; 0.95 to 0.99, substantial; and ≥0.99: almost perfect [11].

## Results

From the initial search of the PubMed archive, 76 studies were retrieved (S1 Table), of which 10 met our pre-specified criteria and were compared with estimates from Livingstone. These comparator studies are detailed in Table 1. S2 Table summarises the reasons why studies were eliminated.

**Table 1:** List of selected comparator studies.

Study Title	Disease or condition	Data source	Codes listed?	Years	Incidence	Prevalence
Prevalence and incidence of neuromuscular conditions in the UK between 2000 and 2019: A retrospective study using primary care data [12]	Inflammatory myopathy, muscular dystrophy, Charcot-Marie-Tooth, Guillain-Barré syndrome, myasthenia gravis, motor neurone disease	CPRD GOLD and Aurum	Yes	2000–2019	Yes*	Yes (Point)*
Incidence, prevalence, and survival of patients with idiopathic pulmonary fibrosis in the UK [26]	Idiopathic pulmonary fibrosis	CPRD GOLD	Yes	2000–2010	Yes	Yes (Point)
Prevalence, healthcare resource utilization and mortality of Lennox-Gastaut syndrome: retrospective linkage cohort study [27]	Lennox-Gastaut syndrome	CPRD GOLD	Yes	2017–2017	No	Yes (Period)
Incidence, prevalence and mortality of patients with psoriasis: a U.K. population-based cohort study [28]	Psoriasis	CPRD GOLD	Yes	1999–2013	Yes	Yes (Period)
Population trends in the 10-year incidence and prevalence of diabetic retinopathy in the UK: a cohort study in the Clinical Practice Research Datalink 2004–2014 [14]	Diabetic retinopathy	CPRD GOLD	Yes	2004–2014	Yes**	Yes (Period)**
Trends in optic neuritis incidence and prevalence in the UK and association with systemic and neurologic disease [15]	Optic neuritis	THIN	Yes	1995–2019	Yes	Yes (Point)
A descriptive epidemiological study of the incidence of newly diagnosed Lyme disease cases in a UK primary care cohort, 1998–2016 [20]	Lyme disease	THIN	Yes	1998–2016	Yes	No
Trends in incidence and prevalence of osteoarthritis in the United Kingdom: findings from the Clinical Practice Research Datalink (CPRD) [19]	Osteoarthritis	CPRD GOLD	Yes	1997–2017	Yes***	Yes (Point)***
The incidence, prevalence, and survival of systemic sclerosis in the UK Clinical Practice Research Datalink [29]	Systemic sclerosis	CPRD GOLD	No	1994–2013	Yes	Yes (Point)
Incidence of nonvalvular atrial fibrillation and oral anticoagulant prescribing in England, 2009 to 2019: A cohort study [13]	Non-valvular atrial fibrillation	CPRD GOLD and Aurum	Yes	2009–2019	Yes	No

*Standardised to 2019 population

**Standardised to 2014 population, only patient aged over 12 included

***Only patients aged over 20 included

### Incidence data from published studies

Together, the 10 published comparator studies reported estimates of incidence for 14 diseases. The most recent annual incidence values from these studies, along with the estimates produced by Livingstone, are shown in Table 2. Incidence rates were presented with denominators depending on the magnitude of the numerator. The incidence values produced by Livingstone for the 14 diseases ranged from 0.73 per 1,000,000 person-years to 7.14 per 1,000 person-years. For the comparative studies, the range was 1.30 per 100,000 to 6.78 per 1,000 person-years.

**Table 2:** Most recent annual incidence and prevalence values from published studies and from Livingstone.

Disease or condition	Incidence				Prevalence			
	Year	Denominator (person-years)	Published value	Livingstone	Year	Denominator (person-years)	Published value	Livingstone
Charcot-Marie-Tooth disease	2019	100,000	1.3	1.6	2019	100,000	29.5	27.7
Diabetic retinopathy	2014	1,000	1.68	1.90	2014	1,000	22.01	20.03
Guillain-Barré syndrome	2019	100,000	1.5	1.6	2019	100,000	40.1	40.0
Idiopathic pulmonary fibrosis	2012	100,000	2.26	2.86	2012	100,000	10.57	10.32
Inflammatory myopathy	2019	100,000	1.4	1.4	2019	100,000	25.0	24.5
Lyme disease	2016	100,000	4.89	6.33	2019	1,000,000	Not available	17.28
Lennox-Gastaut syndrome	2017	1,000,000	Not available	0.732	2017	10,000	0.289	0.167
Motor neurone disease	2019	100,000	3.2	3.3	2019	100,000	12.6	12.3
Muscular dystrophy	2019	100,000	1.3	1.6	2019	100,000	29.5	31.1
Myasthenia gravis	2019	100,000	2.5	2.5	2019	100,000	33.7	32.9
Nonvalvular atrial fibrillation	2019	10,000	25.5	25.6	2019	100	Not available	2.02
Optic neuritis	2018	100,000	3.51	3.88	2018	100,000	114.8	98.4
Osteoarthritis	2017	1,000	6.78	7.14	2017	100	10.77	10.61
Systemic sclerosis	2013	1,000,000	18.6	21.5	2013	1,000,000	307	305

### Incidence concordance

**Fig 1:**
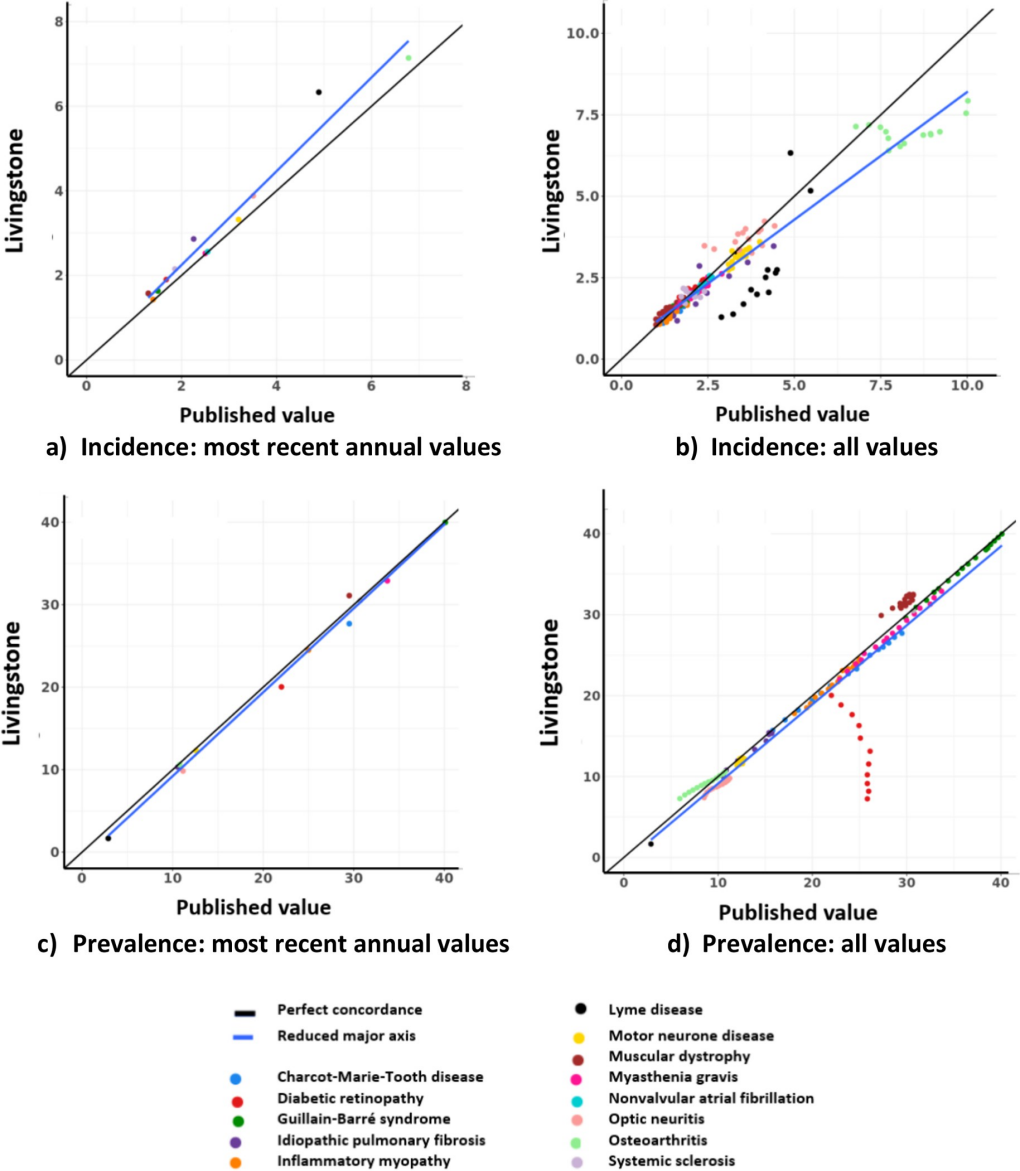
Correlation of incidence and prevalence values for the most recent year available and for all years available.

The concordance between the most recent annual incidence values reported by the comparator studies and those produced by Livingstone was 0.96 (95% CI: 0.89–0.98; Fig 1A). The concordance between all of the annual incidence values reported by the comparator studies and their equivalent values from Livingstone is illustrated in Fig 1B. The overall concordance for all reported values was 0.93 (0.91–0.94). Two studies published gender-specific incidence. The overall gender-specific concordance was 0.93 (0.88–0.96). For individual diseases, concordances between incidence values ranged from 0.95 (0.86–0.98) for diabetic retinopathy to -0.04 (-0.58–0.52) for systemic sclerosis (Table 3). The study of Lennox-Gastaut syndrome reported no values for incidence.

**Table 3:** Condition-specific incidence and prevalence concordance.

Disease or condition	Incidence	Prevalence
	Concordance (95% CIs)	
Charcot-Marie-Tooth disease	0.61 (0.27–0.82)	0.96 (0.92–0.98)
Diabetic retinopathy	0.95 (0.86–0.98)	-0.06 (-0.13–0.00)
Guillain-Barré syndrome	0.80 (0.58–0.91)	1.00 (0.99–1.00)
Idiopathic pulmonary fibrosis	0.82 (0.49–0.95)	0.98 (0.94–1.00)
Inflammatory myopathy	0.86 (0.70–0.94)	0.95 (0.89–0.98)
Lyme disease	0.36 (0.09–0.57)	Not available
Motor neurone disease	0.52 (0.25–0.72)	0.20 (0.02–0.37)
Muscular dystrophy	0.78 (0.56–0.89)	0.24 (0.08–0.38)
Myasthenia gravis	0.89 (0.75–0.95)	0.97 (0.94–0.99)
Nonvalvular atrial fibrillation	0.91 (0.75–0.97)	Not available
Optic neuritis	0.46 (0.07–0.73)	0.47 (0.25–0.64)
Osteoarthritis	0.12 (-0.03–0.27)	0.86 (0.75–0.92)
Systemic sclerosis	-0.04 (-0.58–0.52)	Not available

### Prevalence data from published studies

The 10 comparator studies reported estimates of prevalence for 12 diseases.

Table 2 shows the most recent annual prevalence rates from these studies along with the estimates produced by Livingstone. Estimates of prevalence generated by Livingstone ranged from 0.167 per 10,000 person-years to 10.61 per 100 person-years. For the published studies, the range was 0.289 per 10,000 person-years to 10.77 per 100 person-years.

### Prevalence concordance

The concordance between the final annual prevalence value reported in each comparator study and that produced by Livingstone was 1.00 (0.99–1.00; Fig 1C). Two studies also published gender-specific prevalence, where the concordance was 0.95 (0.90–0.98). For all annual prevalence values reported in each study, the concordance was 0.93 (0.90–0.95; Fig 1D). It was evident that the estimates for diabetic retinopathy were poorly correlated. When these values were removed, the concordance was then 0.99 (0.99–1:00). For each disease individually, the concordance ranged from 1.00 (0.99–1.00) for Guillain-Barré syndrome to -0.06 (-0.13–0.00) for diabetic retinopathy (Table 3). The studies of systemic sclerosis and Lennox-Gastaut syndrome reported only one value for each and, therefore, concordance could not be determined. No values of NVAF were reported for prevalence, so no concordance was calculated.

## Discussion

This study compared incidence and prevalence estimates for a range of diseases derived from published studies with those generated automatically by Livingstone, an online, cloud-based, analytical platform. For comparison of estimates for the most recent years the concordance of prevalence was near perfect (1.00), and for incidence it was substantial (0.96).

Whilst both sets of estimates were derived from routine NHS data, they did not necessarily use the same data sources, thus we did not anticipate replicating published estimates precisely. The estimates derived from Livingstone were based on the combined CPRD Aurum (EMIS) and GOLD (Vision) datasets, but only two of the comparator studies used the same combined data [12,13]. The remaining studies used either CPRD GOLD alone or THIN, with both sources derived from primary care practices using Vision software. Compared with those studies that did use CPRD Aurum and GOLD in combination, our estimates were derived from a later build and had substantially larger versions of the data source. In addition, there were some differences in the methods of calculation. For chronic conditions, Livingstone calculated point prevalence, whereas two of the comparator studies [14,15] calculated period prevalence, which would produce systematically higher estimates. Two studies also standardised their annual estimates to the most recent year, and here again one would expect a difference from estimates derived automatically from Livingstone [12,14].

As can be seen from the S2 and S3 Tables, the majority of diseases studied reported an increase in both incidence and prevalence over time. Secular changes in the reported prevalence and incidence of a disease may be due to a genuine increase or be due to differences in case ascertainment. In addition, when estimates are derived from routine data sources, an observed increase may be an artefact of an increase in the recording of diagnoses on electronic healthcare systems. This has been observed in CPRD [15]. Computerised systems allow users to enter diagnoses as free text and/or by entering clinical codes, so changes in the proportional use of these alternative methods of data entry over time will impact apparent prevalence estimates derived solely from clinical codes. Equally, letters from secondary care that contain diagnostic information can be scanned into the patient record, or the practice could extract data from them and enter clinical codes into the electronic record. Increased recording of clinical coding of electronic records was incentivised by the Quality and Outcomes Framework (QOF), introduced in 2003 [16].

It was therefore important in establishing validity that we should only compare estimates for directly comparable years. The estimate of prevalence for the most recent years had a concordance of 1.00 and, as can be seen from Table 3, the individual estimates were broadly comparable. When comparing the prevalence values of individual diseases over time, however, there was less concordance. For diabetic retinopathy, the concordance for values from 2004 to 2014 was poor (-0.06). Mathur and colleagues reported a reasonably stable prevalence of 25.83 per 10,000 people in 2004 and then 22.01 in 2014 [14], but values from Livingstone showed a lower starting value but a larger increase from 7.25 to 20.03 per 10,000 people, respectively. During this period, there was a greater awareness of the need for systematic screening for diabetic retinopathy, with a national screening scheme introduced in 2005 [17]. It has been reported that the prevalence of recorded diabetes also increased dramatically from 2003 due to factors such as greater awareness of the condition [18,19]. Therefore, we question the reliability of the data provided in the published study.

As described above, the estimates from two comparator studies were age- and gender-standardised to an index year. This included the six neuromuscular conditions studied by Carey and colleagues [12], which explains the poorer concordance with Livingstone for these individual diseases by year, while the estimates derived for the index year (2019) are highly concordant. Due to the relative consistency of the estimates for certain conditions over the study period, the underlying bivariate distribution was heavy-tailed and in these circumstances Lin’s CCC was less robust. This can be observed with systemic sclerosis which had a concordance of -0.04 despite the annual estimates being broadly similar. The greatest difference, reported in 2004, was 1.79 per 100,000 in the published source compared with 2.17 per 100,000 from Livingstone.

The lower concordance for incidence in the most recent year (CCC = 0.96) was partly expected because the calculation of incidence has more scope for variability. For example, it is necessary to choose a sufficient wash-in period in order to maximise the number of truly incident cases that can be reliably designated as such. Equally, it is more difficult to define cases of an acute disease than of a chronic disease. In this study, only one acute condition, Lyme disease, was retrieved based on our selection criteria. However, Tulloch and colleagues [20] only considered first events, so in effect their calculation method was the same as for a chronic condition, since patients with multiple, discrete events were only included once. Consequently, the concordance between the two sets of estimates was poor (CCC = 0.36). In addition, the study by Tulloch and colleagues was conducted using data from THIN [7], so differences in clinical code lists may have also contributed to these differences. These examples help explain why some differences between the concordance of the findings were expected. More importantly. though, it provides an important illustration of the incentive to use epidemiological methods that are standardised, replicable and validated. This has not, until now, been possible.

The nature of the UK health system means that we have detailed longitudinal healthcare data for a non-selective, large proportion of the population that is readily accessible for analysis. Whilst not entirely unique, this is unusual. This isn’t so for insurance claims data or Medicare records in the USA, for example. These alternative healthcare systems produce data that are from selective population groups, which means that these epidemiological metrics require further modelling to estimate overall population values. With regards to determination of reliable descriptive epidemiology, Livingstone should work in most circumstances where data are comprehensive, appropriate denominators exist or they have been estimated in a deliberate statistical exercise. However, there will be instances where this will not be the case and Livingstone could then be used to generate instant curated data to rapidly carry out these statistical modelling exercises. With regards to other health common data models such as OMOP, Livingstone is optimised to use detailed, linked NHS data from multiple records systems. The use of OMOP would lose a lot of important data granularity so we have avoided using this procedure. However, a filter could be easily applied, and the platform would run as normal.

Other platforms exist that expedite healthcare data analysis such as the *Aetion Evidence Platform* [21], *Instant Health Data Analytics* from Panalgo [22], and *Dexter* from investigators at Birmingham University [23]. These alternative platforms differ markedly from Livingstone in that they expedite health research by more quickly producing curated data for further analysis. Livingstone produces complete analyses in only a few minutes. A similar study protocol by the Dexter group was reported in March, 2021 [24], and first published in May, 2022 [25].

There are technical challenges in undertaking epidemiological studies of this nature using NHS data. An automated analytical system must be valid, reliable, rapid, reproducible, scalable, and ideally user-friendly so that the platform can be used by as wide an audience as possible. The potential utility of such a system is exemplified by the pandemic. COVID-19 showed the utility of rapid health intelligence in public health protection. If it had been available, our automated analytical platform might have been of considerable value in monitoring the progression of the epidemic in the UK and, more importantly, in evaluating and monitoring the impact that reduced access to healthcare had and is having on every other disease. The most recent cancer registry statistics for England are available for 2019 and thus provide no insight into the acute problem of the pandemic.

In summary, we have, for the first time, developed an automated system to rapidly and reliably determine the descriptive epidemiology of any disease or condition–and also of any operative procedure, drug exposure or selected phenotype. For the first time, we now have a mechanism to rapidly produce standardised descriptive epidemiology from routine healthcare data.

## Supporting information

S1 Table Summary of the reasons why candidate studies were eliminated. (DOCX)

S2 Table Annual incidence values from published studies and Livingstone. (DOCX)

S3 Table Annual prevalence values from published studies and Livingstone. (DOCX)
