# Correction: Real-world evidence from the first online healthcare analytics platform—Livingstone: Validation of its descriptive epidemiology module

**DOI:** 10.1371/journal.pdig.0001095

**Published:** 2025-11-17

**Authors:** The PLOS Digital Health Editors

After this article [1] was published, concerns were raised about an undisclosed competing interest, namely that the Livingstone analytical platform is owned by the company Human Data Sciences, to which the authors are affiliated.

The article’s competing interest statement is updated to: “The tool evaluated in this study, Livingstone, is a proprietary analytical platform owned and marketed by Human Data Sciences. As is indicated in the listed affiliations, all authors are or were full time employees at Human Data Sciences; some authors hold shares or share options as part of their employment.”

PLOS conducted a post-publication editorial assessment to evaluate whether this disclosure would have affected the article’s peer review outcome. In the post-publication review, *PLOS Digital Health* Editors raised reporting concerns pertaining to aspects of the article’s abstract, discussion and conclusions, but they advised that the undisclosed competing interest did not impact the article’s editorial decision.

Additionally, the ethics approval number reported in [1] is linked to a different project on the CPRD website [2]. The authors confirmed that the correct approval number is reported in [1], and a CPRD representative confirmed that the information on the CPRD website was incorrect as of 24 January 2025.
