# Supplementary material for: Real-world evidence from the first online healthcare analytics platform—Livingstone. Validation of its descriptive epidemiology module
Source: PLOS Digit Health. 2023 Jul 25;2(7):e0000310. doi: 10.1371/journal.pdig.0000310 (PMC10368254; doi:10.1371/journal.pdig.0000310)
Supplement: S1 Table — (DOCX) [file pdig.0000310.s001.docx]

**S1 Table | Summary of the reasons why candidate studies were eliminated**

| **Study Title** | **Initial check** |
| --- | --- |
| Trends in the annual consultation incidence and prevalence of low back pain and osteoarthritis in England from 2000 to 2019: comparative estimates from two clinical practice databases | Uses another denominator |
| Prevalence and patient outcomes of adult primary hypercholesterolemia and dyslipidemia in the UK: longitudinal retrospective study using a primary care dataset from 2009 to 2019 | Uses linked HES data |
| Analysis of incidence of motor neuron disease in England 1998-2019: use of three linked datasets | Uses linked HES data |
| Incidence, prevalence, and natural history of primary sclerosing cholangitis in the United Kingdom | Uses linked HES data |
| Measuring prevalence and incidence of chronic conditions in claims and electronic health record databases | Uses another denominator |
| Prevalence of anal fistula in the United Kingdom | Outcome of disease |
| Exploration of trends in the incidence and prevalence of childhood maltreatment and domestic abuse recording in UK primary care: a retrospective cohort study using 'the health improvement network' database | Uses another denominator |
| Trends in the incidence of chronic fatigue syndrome and fibromyalgia in the UK, 2001-2013: a Clinical Practice Research Datalink study | Uses another denominator |
| Trends in the contemporary incidence of colorectal cancer and patient characteristics in the United Kingdom: a population-based cohort study using The Health Improvement Network | Population over 40 |
| Time trends in peripheral artery disease incidence, prevalence and secondary preventive therapy: a cohort study in The Health Improvement Network in the UK | Population over 50 |
| Incidence of direct oral anticoagulant use in patients with nonvalvular atrial fibrillation and characteristics of users in 6 European countries (2008-2015): A cross-national drug utilization study | Outcome of disease |
| Incidence of shoulder dislocations in the UK, 1995-2015: a population-based cohort study | Looks over whole period |
| Ethnic differences in the incidence of clinically diagnosed influenza: an England population-based cohort study 2008-2018 | Split by ethnicity |
| Socioeconomic deprivation and regional variation in Hodgkin's lymphoma incidence in the UK: a population-based cohort study of 10 million individuals | Looks over whole period |
| Temporal trends and patterns in heart failure incidence: a population-based study of 4 million individuals | Uses linked HES data |
| The incidence and healthcare costs of persistent postoperative pain following lumbar spine surgery in the UK: a cohort study using the Clinical Practice Research Datalink (CPRD) and Hospital Episode Statistics (HES) | Uses linked HES data |
| Temporal trends in incidence, prevalence, and mortality of atrial fibrillation in primary care | Uses linked HES data |
| Secular trends in fracture incidence in the UK between 1990 and 2012 | Over 50's |
| Linked hospital and primary care database: Analysis of the incidence and impact of psychiatric morbidity following gastrointestinal cancer surgery in England | Uses linked HES data |
| Quality of recording of diabetes in the UK: how does the GP's method of coding clinical data affect incidence estimates? Cross-sectional study using the CPRD database | No comparable outputs |
| Trends in the prevalence, incidence and surgical management of carpal tunnel syndrome between 1993 and 2013: an observational analysis of UK primary care records | Uses treatment as diagnosis |
| Incidence of Lyme disease in the UK: a population-based cohort study | Identified with test |
| Clustering of physical health multimorbidity in people with severe mental illness: An accumulated prevalence analysis of United Kingdom primary care data | Results over whole period |
| Development and validation of clinical prediction models for breast cancer incidence and mortality: a protocol for a dual cohort study | No comparable outputs |
| Incidence and prevalence of juvenile idiopathic arthritis in the United Kingdom, 2000–2018: results from the Clinical Practice Research Datalink | Juvenile population |
| One- and 2-year incidence of osteoporotic fracture: a multi-cohort observational study using routinely collected real-world data | Outcome of disease |
| Temporal trends in annual incidence rates for psychiatric disorders and self-harm among children and adolescents in the UK, 2003-2018 | Juvenile population |
| Impact of multiple cardiovascular medications on mortality after an incidence of ischemic stroke or transient ischemic attack | Looks at mortality |
| Incidence and prevalence of primary care antidepressant prescribing in children and young people in England, 1998–2017: A population-based cohort study | Juvenile population |
| Incidence, risk factors, and health service burden of sequelae of campylobacter and non-typhoidal salmonella infections in England, 2000-2015: A retrospective cohort study using linked electronic health records | Uses linked HES data |
| Ethnic differences in the prevalence of Type 2 Diabetes diagnoses in the UK: cross-sectional analysis of the Health Improvement Network primary care database | London-specific |
| Temporal trends in incidence of Rolandic epilepsy, prevalence of comorbidities and prescribing trends: birth cohort study | Juvenile population |
| Prevalence of established cardiovascular disease in patients with Type 2 Diabetes Mellitus in the UK | Outcome of disease |
| Incidence of type II diabetes in chronic obstructive pulmonary disease: a nested case–control study | Outcome of disease |
| Trends in the incidence of testing for vitamin D deficiency in primary care in the UK: a retrospective analysis of The Health Improvement Network (THIN), 2005–2015 | Identified by test |
| Prevalence of maternal mental illness among children and adolescents in the UK between 2005 and 2017: a national retrospective cohort analysis | Juvenile population |
| Prevalence of comorbid mental and physical illnesses and risks for self-harm and premature death among primary care patients diagnosed with fatigue syndromes | Outcome of disease |
| Incidence of type 2 diabetes mellitus in men receiving steroid 5α-reductase inhibitors: population based cohort study | Outcome of disease |
| Prevalence of atopic eczema among patients seen in primary care: data from The Health Improvement Network | Looks over whole period |
| Incidence and risk of celiac disease after type 1 diabetes: A population-based cohort study using the health improvement network database | Outcome of disease |
| Association of guideline and policy changes with incidence of lifestyle advice and treatment for uncomplicated mild hypertension in primary care: a longitudinal cohort study in the Clinical Practice Research Datalink | Identified by test |
| Incidence of subsequent fractures in the UK between 1990 and 2012 among individuals 50 years or older | Outcome of disease |
| Antidepressant utilisation and incidence of weight gain during 10 years' follow-up: population based cohort study | Outcome of disease |
| Cataract in patients with diabetes mellitus-incidence rates in the UK and risk factors | Outcome of disease |
| Increased prevalence of polyneuropathy in Parkinson's disease patients: an observational study | Outcome of disease |
| Estimating the prevalence of generalized and partial lipodystrophy: findings and challenges | Results over whole period |
| Incidence of live-attenuated influenza vaccine administration beyond expiry date in children and adolescents aged 2-17 years in the UK: a population-based cohort study | Juvenile population |
| Increased prevalence of HCV and hepatic decompensation in adults with psoriasis: a population-based study in the United Kingdom | Outcome of disease |
| Prevalence and incidence trends for diagnosed prescription opioid use disorders in the United Kingdom | Outcome of disease |
| Incidence of intracranial bleeds in new users of low-dose aspirin: a cohort study using The Health Improvement Network | Drug outcome |
| Antiepileptic drugs prescribed in pregnancy and prevalence of major congenital malformations: comparative prevalence studies | Drug outcome |
| Validity of estimated prevalence of decreased kidney function and renal replacement therapy from primary care electronic health records compared with national survey and registry data in the United Kingdom | Identified by test |
| Incidence and management of cardiovascular risk factors in psoriatic arthritis and rheumatoid arthritis: a population-based study | Outcome of disease |
| Latest trends in ADHD drug prescribing patterns in children in the UK: prevalence, incidence and persistence | Juvenile population |
| Differing patterns in thermal injury incidence and hospitalisations among 0-4 year old children from England | Juvenile population |
| Impact of hyperkalaemia definition on incidence assessment: implications for epidemiological research based on a large cohort study in newly diagnosed heart failure patients in primary care | Outcome of disease |
| Incidence of adult Huntington's disease in the UK: a UK-based primary care study and a systematic review | Looks over whole period |
| Kawasaki disease incidence in children and adolescents: an observational study in primary care | Juvenile population |
| A cohort study of trends in the prevalence of pregestational diabetes in pregnancy recorded in UK general practice between 1995 and 2012 | Uses another denominator |
| Is environmental radon gas associated with the incidence of neurodegenerative conditions? A retrospective study of multiple sclerosis in radon affected areas in England and Wales | No comparable outputs |
| Trends in incidence, prevalence and prescribing in type 2 diabetes mellitus between 2000 and 2013 in primary care: a retrospective cohort study | No comparable outputs |
| Incidence of osteoporosis and fragility fractures in asthma: a UK population-based matched cohort study | Outcome of disease |
| The incidence and prevalence of systemic lupus erythematosus in the UK, 1999-2012 | HES |
| The incidence of fractures at various sites in newly treated patients with type 2 diabetes mellitus | Outcome of disease |
| Characterising the background incidence rates of adverse events of special interest for covid-19 vaccines in eight countries: multinational network cohort study | Outcome of disease |
| Prevalence and healthcare resource utilization of patients with Dravet syndrome: retrospective linkage cohort study | Uses linked HES data |
