# Supplementary material for: Real-world evidence from the first online healthcare analytics platform—Livingstone. Validation of its descriptive epidemiology module
Source: PLOS Digit Health. 2023 Jul 25;2(7):e0000310. doi: 10.1371/journal.pdig.0000310 (PMC10368254; doi:10.1371/journal.pdig.0000310)
Supplement: S2 Table — (DOCX) [file pdig.0000310.s002.docx]

**S2 Table | Annual incidence values from published studies and Livingstone**

| **Disease or condition** | **Year** | **Reported incidence** | **Livingstone Incidence** | **Denominator (population/ person-years)** |
| --- | --- | --- | --- | --- |
| Charcot-Marie-Tooth disease | 2004 | 1.60 | 1.48 | 100,000 |
| Charcot-Marie-Tooth disease | 2005 | 1.50 | 1.36 | 100,000 |
| Charcot-Marie-Tooth disease | 2006 | 1.70 | 1.47 | 100,000 |
| Charcot-Marie-Tooth disease | 2007 | 1.80 | 1.62 | 100,000 |
| Charcot-Marie-Tooth disease | 2008 | 1.50 | 1.33 | 100,000 |
| Charcot-Marie-Tooth disease | 2009 | 1.30 | 1.24 | 100,000 |
| Charcot-Marie-Tooth disease | 2010 | 1.40 | 1.33 | 100,000 |
| Charcot-Marie-Tooth disease | 2011 | 1.30 | 1.35 | 100,000 |
| Charcot-Marie-Tooth disease | 2012 | 1.20 | 1.11 | 100,000 |
| Charcot-Marie-Tooth disease | 2013 | 1.40 | 1.36 | 100,000 |
| Charcot-Marie-Tooth disease | 2014 | 1.30 | 1.30 | 100,000 |
| Charcot-Marie-Tooth disease | 2015 | 1.40 | 1.30 | 100,000 |
| Charcot-Marie-Tooth disease | 2016 | 1.50 | 1.37 | 100,000 |
| Charcot-Marie-Tooth disease | 2017 | 1.60 | 1.49 | 100,000 |
| Charcot-Marie-Tooth disease | 2018 | 1.40 | 1.33 | 100,000 |
| Charcot-Marie-Tooth disease | 2019 | 1.30 | 1.57 | 100,000 |
| Diabetic retinopathy | 2004 | 1.21 | 1.39 | 1,000 |
| Diabetic retinopathy | 2005 | 1.34 | 1.41 | 1,000 |
| Diabetic retinopathy | 2006 | 1.55 | 1.59 | 1,000 |
| Diabetic retinopathy | 2007 | 1.82 | 1.85 | 1,000 |
| Diabetic retinopathy | 2008 | 2.07 | 2.12 | 1,000 |
| Diabetic retinopathy | 2009 | 2.32 | 2.36 | 1,000 |
| Diabetic retinopathy | 2010 | 2.35 | 2.42 | 1,000 |
| Diabetic retinopathy | 2011 | 2.39 | 2.44 | 1,000 |
| Diabetic retinopathy | 2012 | 2.14 | 2.21 | 1,000 |
| Diabetic retinopathy | 2013 | 1.93 | 2.16 | 1,000 |
| Diabetic retinopathy | 2014 | 1.68 | 1.9 | 1,000 |
| Guillain-Barré syndrome | 2004 | 1.50 | 1.48 | 100,000 |
| Guillain-Barré syndrome | 2005 | 1.50 | 1.61 | 100,000 |
| Guillain-Barré syndrome | 2006 | 1.50 | 1.44 | 100,000 |
| Guillain-Barré syndrome | 2007 | 1.50 | 1.46 | 100,000 |
| Guillain-Barré syndrome | 2008 | 1.90 | 1.77 | 100,000 |
| Guillain-Barré syndrome | 2009 | 1.80 | 1.72 | 100,000 |
| Guillain-Barré syndrome | 2010 | 1.60 | 1.61 | 100,000 |
| Guillain-Barré syndrome | 2011 | 1.70 | 1.64 | 100,000 |
| Guillain-Barré syndrome | 2012 | 1.70 | 1.64 | 100,000 |
| Guillain-Barré syndrome | 2013 | 1.80 | 1.72 | 100,000 |
| Guillain-Barré syndrome | 2014 | 1.50 | 1.48 | 100,000 |
| Guillain-Barré syndrome | 2015 | 1.70 | 1.68 | 100,000 |
| Guillain-Barré syndrome | 2016 | 1.80 | 1.68 | 100,000 |
| Guillain-Barré syndrome | 2017 | 1.80 | 1.78 | 100,000 |
| Guillain-Barré syndrome | 2018 | 1.90 | 1.74 | 100,000 |
| Guillain-Barré syndrome | 2019 | 1.50 | 1.63 | 100,000 |
| Idiopathic pulmonary fibrosis | 2004 | 4.40 | 3.47 | 100,000 |
| Idiopathic pulmonary fibrosis | 2005​ | 3.65 | 2.97 | 100,000 |
| Idiopathic pulmonary fibrosis | 2006​ | 3.11 | 2.55 | 100,000 |
| Idiopathic pulmonary fibrosis | 2007​ | 2.47 | 2.03 | 100,000 |
| Idiopathic pulmonary fibrosis | 2008​ | 2.15 | 1.69 | 100,000 |
| Idiopathic pulmonary fibrosis | 2009​ | 1.50 | 1.46 | 100,000 |
| Idiopathic pulmonary fibrosis | 2010​ | 1.61 | 1.18 | 100,000 |
| Idiopathic pulmonary fibrosis | 2011​ | 1.50 | 1.33 | 100,000 |
| Idiopathic pulmonary fibrosis | 2012​ | 2.26 | 2.86 | 100,000 |
| Inflammatory myopathy | 2004 | 1.90 | 1.66 | 100,000 |
| Inflammatory myopathy | 2005 | 1.40 | 1.36 | 100,000 |
| Inflammatory myopathy | 2006 | 1.40 | 1.37 | 100,000 |
| Inflammatory myopathy | 2007 | 1.60 | 1.47 | 100,000 |
| Inflammatory myopathy | 2008 | 1.30 | 1.27 | 100,000 |
| Inflammatory myopathy | 2009 | 1.30 | 1.28 | 100,000 |
| Inflammatory myopathy | 2010 | 1.40 | 1.25 | 100,000 |
| Inflammatory myopathy | 2011 | 1.10 | 1.08 | 100,000 |
| Inflammatory myopathy | 2012 | 1.30 | 1.14 | 100,000 |
| Inflammatory myopathy | 2013 | 1.10 | 1.15 | 100,000 |
| Inflammatory myopathy | 2014 | 1.10 | 1.08 | 100,000 |
| Inflammatory myopathy | 2015 | 1.30 | 1.25 | 100,000 |
| Inflammatory myopathy | 2016 | 1.30 | 1.34 | 100,000 |
| Inflammatory myopathy | 2017 | 1.40 | 1.35 | 100,000 |
| Inflammatory myopathy | 2018 | 1.10 | 1.19 | 100,000 |
| Inflammatory myopathy | 2019 | 1.40 | 1.43 | 100,000 |
| Lyme disease | 2004 | 2.89 | 1.29 | 100,000 |
| Lyme disease | 2005 | 3.23 | 1.38 | 100,000 |
| Lyme disease | 2006 | 3.53 | 1.69 | 100,000 |
| Lyme disease | 2007 | 3.92 | 1.99 | 100,000 |
| Lyme disease | 2008 | 4.26 | 2.05 | 100,000 |
| Lyme disease | 2009 | 4.50 | 2.74 | 100,000 |
| Lyme disease | 2010 | 4.23 | 2.74 | 100,000 |
| Lyme disease | 2011 | 4.17 | 2.51 | 100,000 |
| Lyme disease | 2012 | 3.75 | 2.13 | 100,000 |
| Lyme disease | 2013 | 4.46 | 2.65 | 100,000 |
| Lyme disease | 2014 | 3.36 | 3.25 | 100,000 |
| Lyme disease | 2015 | 5.47 | 5.17 | 100,000 |
| Lyme disease | 2016 | 4.89 | 6.33 | 100,000 |
| Motor neurone disease | 2004 | 3.20 | 3.00 | 100,000 |
| Motor neurone disease | 2005 | 3.50 | 3.14 | 100,000 |
| Motor neurone disease | 2006 | 3.20 | 2.80 | 100,000 |
| Motor neurone disease | 2007 | 3.10 | 2.74 | 100,000 |
| Motor neurone disease | 2008 | 3.10 | 2.91 | 100,000 |
| Motor neurone disease | 2009 | 3.40 | 3.33 | 100,000 |
| Motor neurone disease | 2010 | 3.70 | 3.42 | 100,000 |
| Motor neurone disease | 2011 | 3.30 | 3.13 | 100,000 |
| Motor neurone disease | 2012 | 3.60 | 3.16 | 100,000 |
| Motor neurone disease | 2013 | 3.70 | 3.38 | 100,000 |
| Motor neurone disease | 2014 | 3.40 | 3.19 | 100,000 |
| Motor neurone disease | 2015 | 4.00 | 3.60 | 100,000 |
| Motor neurone disease | 2016 | 3.50 | 3.30 | 100,000 |
| Motor neurone disease | 2017 | 3.40 | 3.07 | 100,000 |
| Motor neurone disease | 2018 | 3.60 | 3.36 | 100,000 |
| Motor neurone disease | 2019 | 3.20 | 3.32 | 100,000 |
| Muscular dystrophy | 2004 | 1.80 | 1.89 | 100,000 |
| Muscular dystrophy | 2005 | 1.60 | 1.74 | 100,000 |
| Muscular dystrophy | 2006 | 1.70 | 1.67 | 100,000 |
| Muscular dystrophy | 2007 | 1.20 | 1.46 | 100,000 |
| Muscular dystrophy | 2008 | 1.40 | 1.60 | 100,000 |
| Muscular dystrophy | 2009 | 1.30 | 1.51 | 100,000 |
| Muscular dystrophy | 2010 | 1.30 | 1.41 | 100,000 |
| Muscular dystrophy | 2011 | 1.50 | 1.58 | 100,000 |
| Muscular dystrophy | 2012 | 1.40 | 1.48 | 100,000 |
| Muscular dystrophy | 2013 | 1.00 | 1.23 | 100,000 |
| Muscular dystrophy | 2014 | 1.00 | 1.06 | 100,000 |
| Muscular dystrophy | 2015 | 1.10 | 1.21 | 100,000 |
| Muscular dystrophy | 2016 | 1.10 | 1.18 | 100,000 |
| Muscular dystrophy | 2017 | 1.10 | 1.39 | 100,000 |
| Muscular dystrophy | 2018 | 1.10 | 1.22 | 100,000 |
| Muscular dystrophy | 2019 | 1.30 | 1.58 | 100,000 |
| Myasthenia gravis | 2004 | 1.70 | 1.72 | 100,000 |
| Myasthenia gravis | 2005 | 2.00 | 1.93 | 100,000 |
| Myasthenia gravis | 2006 | 2.10 | 1.97 | 100,000 |
| Myasthenia gravis | 2007 | 1.60 | 1.60 | 100,000 |
| Myasthenia gravis | 2008 | 2.40 | 2.22 | 100,000 |
| Myasthenia gravis | 2009 | 2.10 | 2.00 | 100,000 |
| Myasthenia gravis | 2010 | 2.00 | 1.86 | 100,000 |
| Myasthenia gravis | 2011 | 2.00 | 2.06 | 100,000 |
| Myasthenia gravis | 2012 | 2.30 | 2.10 | 100,000 |
| Myasthenia gravis | 2013 | 2.20 | 2.02 | 100,000 |
| Myasthenia gravis | 2014 | 2.50 | 2.26 | 100,000 |
| Myasthenia gravis | 2015 | 2.40 | 2.32 | 100,000 |
| Myasthenia gravis | 2016 | 2.50 | 2.33 | 100,000 |
| Myasthenia gravis | 2017 | 2.30 | 2.27 | 100,000 |
| Myasthenia gravis | 2018 | 2.90 | 2.62 | 100,000 |
| Myasthenia gravis | 2019 | 2.50 | 2.52 | 100,000 |
| Nonvalvular atrial fibrillation | 2009 | 2.08 | 2.02 | 1,000 |
| Nonvalvular atrial fibrillation | 2010 | 2.15 | 2.03 | 1,000 |
| Nonvalvular atrial fibrillation | 2011 | 2.18 | 2.08 | 1,000 |
| Nonvalvular atrial fibrillation | 2012 | 2.31 | 2.23 | 1,000 |
| Nonvalvular atrial fibrillation | 2013 | 2.28 | 2.27 | 1,000 |
| Nonvalvular atrial fibrillation | 2014 | 2.41 | 2.33 | 1,000 |
| Nonvalvular atrial fibrillation | 2015 | 2.59 | 2.48 | 1,000 |
| Nonvalvular atrial fibrillation | 2016 | 2.58 | 2.45 | 1,000 |
| Nonvalvular atrial fibrillation | 2017 | 2.62 | 2.52 | 1,000 |
| Nonvalvular atrial fibrillation | 2018 | 2.57 | 2.51 | 1,000 |
| Nonvalvular atrial fibrillation | 2019 | 2.55 | 2.56 | 1,000 |
| Optic neuritis | 2004 | 3.96 | 3.91 | 100,000 |
| Optic neuritis | 2005 | 4.43 | 4.09 | 100,000 |
| Optic neuritis | 2006 | 4.14 | 4.23 | 100,000 |
| Optic neuritis | 2007 | 4.03 | 3.98 | 100,000 |
| Optic neuritis | 2008 | 3.68 | 3.69 | 100,000 |
| Optic neuritis | 2009 | 3.40 | 3.35 | 100,000 |
| Optic neuritis | 2010 | 3.41 | 3.37 | 100,000 |
| Optic neuritis | 2011 | 3.77 | 3.26 | 100,000 |
| Optic neuritis | 2012 | 4.07 | 3.49 | 100,000 |
| Optic neuritis | 2013 | 3.37 | 3.84 | 100,000 |
| Optic neuritis | 2014 | 3.29 | 3.60 | 100,000 |
| Optic neuritis | 2015 | 2.40 | 3.48 | 100,000 |
| Optic neuritis | 2016 | 3.59 | 4.00 | 100,000 |
| Optic neuritis | 2017 | 2.68 | 3.38 | 100,000 |
| Optic neuritis | 2018 | 3.51 | 3.88 | 100,000 |
| Osteoarthritis | 2004 | 10.02 | 7.93 | 1,000 |
| Osteoarthritis | 2005 | 9.97 | 7.55 | 1,000 |
| Osteoarthritis | 2006 | 9.21 | 6.98 | 1,000 |
| Osteoarthritis | 2007 | 8.95 | 6.89 | 1,000 |
| Osteoarthritis | 2008 | 8.94 | 6.92 | 1,000 |
| Osteoarthritis | 2009 | 8.73 | 6.88 | 1,000 |
| Osteoarthritis | 2010 | 8.18 | 6.62 | 1,000 |
| Osteoarthritis | 2011 | 8.06 | 6.53 | 1,000 |
| Osteoarthritis | 2012 | 7.73 | 6.4 | 1,000 |
| Osteoarthritis | 2013 | 7.72 | 6.78 | 1,000 |
| Osteoarthritis | 2014 | 7.65 | 6.98 | 1,000 |
| Osteoarthritis | 2015 | 7.49 | 7.12 | 1,000 |
| Osteoarthritis | 2016 | 7.16 | 7.19 | 1,000 |
| Osteoarthritis | 2017 | 6.78 | 7.14 | 1,000 |
| Systemic sclerosis | 2004 | 1.79 | 2.17 | 100,000 |
| Systemic sclerosis | 2005 | 2.11 | 1.96 | 100,000 |
| Systemic sclerosis | 2006 | 1.96 | 2.02 | 100,000 |
| Systemic sclerosis | 2007 | 2.12 | 1.91 | 100,000 |
| Systemic sclerosis | 2008 | 1.73 | 1.92 | 100,000 |
| Systemic sclerosis | 2009 | 1.84 | 1.71 | 100,000 |
| Systemic sclerosis | 2010 | 1.87 | 2.09 | 100,000 |
| Systemic sclerosis | 2011 | 2.40 | 2.08 | 100,000 |
| Systemic sclerosis | 2012 | 2.28 | 1.90 | 100,000 |
| Systemic sclerosis | 2013 | 1.86 | 2.15 | 100,000 |
