# Supplementary material for: Real-world evidence from the first online healthcare analytics platform—Livingstone. Validation of its descriptive epidemiology module
Source: PLOS Digit Health. 2023 Jul 25;2(7):e0000310. doi: 10.1371/journal.pdig.0000310 (PMC10368254; doi:10.1371/journal.pdig.0000310)
Supplement: S3 Table — (DOCX) [file pdig.0000310.s003.docx]

**S3 Table | Annual prevalence values from published studies and Livingstone**

| **Disease or condition** | **Year** | **Reported prevalence** | **Livingstone prevalence** | **Denominator (population/ person-years)** |
| --- | --- | --- | --- | --- |
| Charcot-Marie-Tooth disease | 2004 | 15.80 | 15.70 | 100,000 |
| Charcot-Marie-Tooth disease | 2005 | 17.10 | 17.00 | 100,000 |
| Charcot-Marie-Tooth disease | 2006 | 18.50 | 18.20 | 100,000 |
| Charcot-Marie-Tooth disease | 2007 | 20.00 | 19.40 | 100,000 |
| Charcot-Marie-Tooth disease | 2008 | 20.90 | 20.30 | 100,000 |
| Charcot-Marie-Tooth disease | 2009 | 21.90 | 21.20 | 100,000 |
| Charcot-Marie-Tooth disease | 2010 | 22.90 | 22.10 | 100,000 |
| Charcot-Marie-Tooth disease | 2011 | 23.80 | 22.70 | 100,000 |
| Charcot-Marie-Tooth disease | 2012 | 24.70 | 23.30 | 100,000 |
| Charcot-Marie-Tooth disease | 2013 | 25.20 | 24.20 | 100,000 |
| Charcot-Marie-Tooth disease | 2014 | 26.10 | 25.00 | 100,000 |
| Charcot-Marie-Tooth disease | 2015 | 27.00 | 25.70 | 100,000 |
| Charcot-Marie-Tooth disease | 2016 | 27.50 | 26.00 | 100,000 |
| Charcot-Marie-Tooth disease | 2017 | 28.10 | 26.50 | 100,000 |
| Charcot-Marie-Tooth disease | 2018 | 28.70 | 27.20 | 100,000 |
| Charcot-Marie-Tooth disease | 2019 | 29.50 | 27.70 | 100,000 |
| Diabetic retinopathy | 2004 | 25.83 | 7.25 | 1,000 |
| Diabetic retinopathy | 2005 | 25.97 | 8.19 | 1,000 |
| Diabetic retinopathy | 2006 | 25.83 | 9.14 | 1,000 |
| Diabetic retinopathy | 2007 | 25.83 | 10.22 | 1,000 |
| Diabetic retinopathy | 2008 | 25.97 | 11.55 | 1,000 |
| Diabetic retinopathy | 2009 | 26.12 | 13.13 | 1,000 |
| Diabetic retinopathy | 2010 | 25.09 | 14.75 | 1,000 |
| Diabetic retinopathy | 2011 | 24.95 | 16.30 | 1,000 |
| Diabetic retinopathy | 2012 | 24.21 | 17.65 | 1,000 |
| Diabetic retinopathy | 2013 | 23.04 | 18.85 | 1,000 |
| Diabetic retinopathy | 2014 | 22.01 | 20.03 | 1,000 |
| Guillain-Barré syndrome | 2004 | 29.90 | 29.60 | 100,000 |
| Guillain-Barré syndrome | 2005 | 31.00 | 30.91 | 100,000 |
| Guillain-Barré syndrome | 2006 | 32.10 | 31.78 | 100,000 |
| Guillain-Barré syndrome | 2007 | 32.90 | 32.77 | 100,000 |
| Guillain-Barré syndrome | 2008 | 33.40 | 33.20 | 100,000 |
| Guillain-Barré syndrome | 2009 | 34.40 | 34.19 | 100,000 |
| Guillain-Barré syndrome | 2010 | 35.40 | 35.06 | 100,000 |
| Guillain-Barré syndrome | 2011 | 35.90 | 35.72 | 100,000 |
| Guillain-Barré syndrome | 2012 | 36.50 | 36.26 | 100,000 |
| Guillain-Barré syndrome | 2013 | 37.30 | 37.03 | 100,000 |
| Guillain-Barré syndrome | 2014 | 38.40 | 38.01 | 100,000 |
| Guillain-Barré syndrome | 2015 | 38.60 | 38.23 | 100,000 |
| Guillain-Barré syndrome | 2016 | 38.90 | 38.67 | 100,000 |
| Guillain-Barré syndrome | 2017 | 39.30 | 39.10 | 100,000 |
| Guillain-Barré syndrome | 2018 | 39.70 | 39.54 | 100,000 |
| Guillain-Barré syndrome | 2019 | 40.10 | 39.98 | 100,000 |
| Idiopathic pulmonary fibrosis | 2004 | 15.40 | 15.20 | 100,000 |
| Idiopathic pulmonary fibrosis | 2005​ | 15.40 | 15.42 | 100,000 |
| Idiopathic pulmonary fibrosis | 2006​ | 15.71 | 15.35 | 100,000 |
| Idiopathic pulmonary fibrosis | 2007​ | 15.10 | 14.40 | 100,000 |
| Idiopathic pulmonary fibrosis | 2008​ | 13.89 | 13.34 | 100,000 |
| Idiopathic pulmonary fibrosis | 2009​ | 12.08 | 11.95 | 100,000 |
| Idiopathic pulmonary fibrosis | 2010​ | 10.87 | 10.79 | 100,000 |
| Idiopathic pulmonary fibrosis | 2011​ | 10.57 | 9.97 | 100,000 |
| Idiopathic pulmonary fibrosis | 2012​ | 10.57 | 10.32 | 100,000 |
| Inflammatory myopathy | 2004 | 18.10 | 17.80 | 100,000 |
| Inflammatory myopathy | 2005 | 19.40 | 18.50 | 100,000 |
| Inflammatory myopathy | 2006 | 19.80 | 19.00 | 100,000 |
| Inflammatory myopathy | 2007 | 20.30 | 19.80 | 100,000 |
| Inflammatory myopathy | 2008 | 21.00 | 20.30 | 100,000 |
| Inflammatory myopathy | 2009 | 21.70 | 20.80 | 100,000 |
| Inflammatory myopathy | 2010 | 22.10 | 21.30 | 100,000 |
| Inflammatory myopathy | 2011 | 22.70 | 21.70 | 100,000 |
| Inflammatory myopathy | 2012 | 22.90 | 22.20 | 100,000 |
| Inflammatory myopathy | 2013 | 23.20 | 23.10 | 100,000 |
| Inflammatory myopathy | 2014 | 23.80 | 23.30 | 100,000 |
| Inflammatory myopathy | 2015 | 24.30 | 23.50 | 100,000 |
| Inflammatory myopathy | 2016 | 24.50 | 23.90 | 100,000 |
| Inflammatory myopathy | 2017 | 24.80 | 24.10 | 100,000 |
| Inflammatory myopathy | 2018 | 25.00 | 24.30 | 100,000 |
| Inflammatory myopathy | 2019 | 25.00 | 24.50 | 100,000 |
| Lennox-Gastaut syndrome | 2017 | 2.89 | 1.67 | 100,000 |
| Motor neurone disease | 2004 | 12.10 | 11.60 | 100,000 |
| Motor neurone disease | 2005 | 12.50 | 11.80 | 100,000 |
| Motor neurone disease | 2006 | 12.60 | 11.60 | 100,000 |
| Motor neurone disease | 2007 | 12.30 | 11.50 | 100,000 |
| Motor neurone disease | 2008 | 12.00 | 11.40 | 100,000 |
| Motor neurone disease | 2009 | 12.30 | 11.90 | 100,000 |
| Motor neurone disease | 2010 | 12.60 | 12.30 | 100,000 |
| Motor neurone disease | 2011 | 12.60 | 12.30 | 100,000 |
| Motor neurone disease | 2012 | 12.80 | 12.20 | 100,000 |
| Motor neurone disease | 2013 | 12.80 | 12.10 | 100,000 |
| Motor neurone disease | 2014 | 12.60 | 11.80 | 100,000 |
| Motor neurone disease | 2015 | 12.30 | 12.20 | 100,000 |
| Motor neurone disease | 2016 | 12.70 | 12.20 | 100,000 |
| Motor neurone disease | 2017 | 12.70 | 12.00 | 100,000 |
| Motor neurone disease | 2018 | 12.30 | 12.00 | 100,000 |
| Motor neurone disease | 2019 | 12.60 | 12.30 | 100,000 |
| Muscular dystrophy | 2004 | 27.30 | 29.90 | 100,000 |
| Muscular dystrophy | 2005 | 28.50 | 30.80 | 100,000 |
| Muscular dystrophy | 2006 | 29.30 | 31.40 | 100,000 |
| Muscular dystrophy | 2007 | 29.80 | 31.50 | 100,000 |
| Muscular dystrophy | 2008 | 29.80 | 31.90 | 100,000 |
| Muscular dystrophy | 2009 | 30.00 | 32.30 | 100,000 |
| Muscular dystrophy | 2010 | 30.30 | 32.20 | 100,000 |
| Muscular dystrophy | 2011 | 30.30 | 32.50 | 100,000 |
| Muscular dystrophy | 2012 | 30.70 | 32.40 | 100,000 |
| Muscular dystrophy | 2013 | 30.70 | 32.50 | 100,000 |
| Muscular dystrophy | 2014 | 30.60 | 31.80 | 100,000 |
| Muscular dystrophy | 2015 | 30.30 | 31.50 | 100,000 |
| Muscular dystrophy | 2016 | 29.90 | 31.10 | 100,000 |
| Muscular dystrophy | 2017 | 29.40 | 30.80 | 100,000 |
| Muscular dystrophy | 2018 | 29.30 | 31.00 | 100,000 |
| Muscular dystrophy | 2019 | 29.50 | 31.10 | 100,000 |
| Myasthenia gravis | 2004 | 22.90 | 22.00 | 100,000 |
| Myasthenia gravis | 2005 | 23.70 | 23.00 | 100,000 |
| Myasthenia gravis | 2006 | 24.60 | 23.90 | 100,000 |
| Myasthenia gravis | 2007 | 25.20 | 24.40 | 100,000 |
| Myasthenia gravis | 2008 | 25.50 | 25.20 | 100,000 |
| Myasthenia gravis | 2009 | 26.70 | 26.00 | 100,000 |
| Myasthenia gravis | 2010 | 27.60 | 26.70 | 100,000 |
| Myasthenia gravis | 2011 | 27.90 | 27.10 | 100,000 |
| Myasthenia gravis | 2012 | 28.50 | 27.70 | 100,000 |
| Myasthenia gravis | 2013 | 29.20 | 28.40 | 100,000 |
| Myasthenia gravis | 2014 | 30.00 | 29.30 | 100,000 |
| Myasthenia gravis | 2015 | 30.80 | 30.10 | 100,000 |
| Myasthenia gravis | 2016 | 31.40 | 30.80 | 100,000 |
| Myasthenia gravis | 2017 | 32.50 | 31.30 | 100,000 |
| Myasthenia gravis | 2018 | 32.90 | 32.10 | 100,000 |
| Myasthenia gravis | 2019 | 33.70 | 32.90 | 100,000 |
| Optic neuritis | 2004 | 8.53 | 7.39 | 10,000 |
| Optic neuritis | 2005 | 8.65 | 7.73 | 10,000 |
| Optic neuritis | 2006 | 8.82 | 8.06 | 10,000 |
| Optic neuritis | 2007 | 9.14 | 8.36 | 10,000 |
| Optic neuritis | 2008 | 9.42 | 8.53 | 10,000 |
| Optic neuritis | 2009 | 9.84 | 8.72 | 10,000 |
| Optic neuritis | 2010 | 10.02 | 8.84 | 10,000 |
| Optic neuritis | 2011 | 10.23 | 8.98 | 10,000 |
| Optic neuritis | 2012 | 10.46 | 9.06 | 10,000 |
| Optic neuritis | 2013 | 10.75 | 9.26 | 10,000 |
| Optic neuritis | 2014 | 10.94 | 9.44 | 10,000 |
| Optic neuritis | 2015 | 11.07 | 9.55 | 10,000 |
| Optic neuritis | 2016 | 11.14 | 9.69 | 10,000 |
| Optic neuritis | 2017 | 11.24 | 9.79 | 10,000 |
| Optic neuritis | 2018 | 11.14 | 9.84 | 10,000 |
| Osteoarthritis | 2004 | 5.95 | 7.28 | 1,000 |
| Osteoarthritis | 2005 | 6.47 | 7.73 | 1,000 |
| Osteoarthritis | 2006 | 6.92 | 8.06 | 1,000 |
| Osteoarthritis | 2007 | 7.34 | 8.34 | 1,000 |
| Osteoarthritis | 2008 | 7.78 | 8.63 | 1,000 |
| Osteoarthritis | 2009 | 8.23 | 8.91 | 1,000 |
| Osteoarthritis | 2010 | 8.60 | 9.13 | 1,000 |
| Osteoarthritis | 2011 | 9.01 | 9.35 | 1,000 |
| Osteoarthritis | 2012 | 9.40 | 9.52 | 1,000 |
| Osteoarthritis | 2013 | 9.82 | 9.81 | 1,000 |
| Osteoarthritis | 2014 | 10.17 | 10.11 | 1,000 |
| Osteoarthritis | 2015 | 10.50 | 10.32 | 1,000 |
| Osteoarthritis | 2016 | 10.66 | 10.48 | 1,000 |
| Osteoarthritis | 2017 | 10.77 | 10.61 | 1,000 |
| Systemic sclerosis | 2013 | 30.70 | 30.50 | 100,000 |
